# AI feedback-seeking and learning engagement in art education: psychological antecedents and reflective mechanisms

**DOI:** 10.3389/fpsyg.2026.1948880

**Published:** 2026-10-01

**Authors:** Tianbi Hu, Guangyao Li

**Affiliations:** 1 School of Art and Humanities, China Academy of Art, Hangzhou, China; 2 Art Design College, Zhejiang Gongshang University, Hangzhou, China

**Keywords:** AI feedback-seeking, art education, fear of negative evaluation, help-seeking avoidance, openness, reflective learning, task behavioral engagement

## Abstract

**Introduction:**

Generative artificial intelligence provides students with a feedback source that differs from traditional teacher and peer interaction, yet limited research has explained why students actively seek feedback from AI or how such feedback-seeking is associated with subsequent learning engagement. Drawing on transactional stress and coping theory and social cognitive theory, this study examined the psychological antecedents and reflective mechanisms of AI feedback-seeking in art education.

**Methods:**

Two complementary studies were conducted. Study 1 utilized survey data from 315 higher education students with recent experience in art learning and generative AI use to investigate whether a fear of negative evaluation was associated with AI feedback-seeking through interpersonal help-seeking avoidance. Study 2 involved 172 students who completed a standardized AI-supported art-creation task. It examined whether AI feedback-seeking was associated with task behavioral engagement through reflective learning and whether openness strengthened this indirect relationship. The proposed relationships were tested using partial least squares structural equation modeling.

**Results:**

In Study 1, fear of negative evaluation was positively associated with interpersonal help-seeking avoidance, which in turn was associated with greater AI feedback-seeking. The indirect association between fear of negative evaluation and AI feedback-seeking through interpersonal help-seeking avoidance was significant, whereas the direct association was not. In Study 2, AI feedback-seeking was positively associated with reflective learning, which was further associated with task behavioral engagement. Reflective learning significantly mediated the association between AI feedback-seeking and task behavioral engagement. Openness strengthened both the association between AI feedback-seeking and reflective learning and the corresponding indirect association with task behavioral engagement.

**Discussion:**

The findings indicate that avoiding interpersonal help does not necessarily reflect withdrawal from feedback. Under evaluation concerns, students may instead select AI as a feedback source involving less interpersonal exposure. However, seeking AI feedback is not uniformly associated with stronger learning engagement; its relevance depends on whether students reflect on the information obtained and remain receptive to alternative perspectives. The study positions AI feedback-seeking as both a coping-oriented form of feedback-source selection and a learner-initiated resource for reflective engagement in art education.

## 1 Introduction

Feedback plays an important role in art learning and the improvement of creative work, yet it may also evoke concerns and avoidance among students (Smith, 2011; Xu and Wang, 2024). During the processes of conceptualizing, presenting, critiquing, and revising artwork, feedback from teachers and peers can help students identify problems, clarify their creative intentions, and refine their modes of expression (Tam, 2018; Pitt and Carless, 2022). However, because artistic work often embodies personal expression and creative identity in art education, negative evaluations of the work may be interpreted by students as judgments of their creative ability and artistic judgment (Smith, 2011; Halverson et al., 2023; Cleary Rodrigues, 2025). Therefore, art feedback carries both the potential to improve creative work and the risk of threatening students’ self-image and exposing them to social-evaluative pressure. Under these conditions, students may simultaneously need external feedback to improve their work and feel reluctant to expose underdeveloped ideas or unfinished work to evaluation by teachers or peers.

The emergence of generative artificial intelligence has provided students with a new route to feedback that differs from traditional interpersonal interaction. AI can offer immediate evaluation, explanations, and suggestions for improvement based on students’ existing work, while also allowing them to ask repeated follow-up questions with relatively low barriers (Henderson et al., 2026; Schmitt-Fumian et al., 2025). Compared with directly revealing difficulties to teachers or peers, asking AI for feedback generally does not require students to publicly expose their uncertainty in front of significant others (Henderson et al., 2026; Zhan et al., 2025; Reinhardt et al., 2025). Students who fear negative evaluation may not cease seeking feedback altogether, but may instead be more inclined to use feedback sources that involve less interpersonal exposure. Accordingly, students who are reluctant to seek feedback from teachers or peers may still obtain diagnostic and improvement-oriented information about their work from AI. This study defines such behavior as AI feedback-seeking, namely, students’ active requests to AI for evaluation, problem diagnosis, clarification, or suggestions for improving their existing ideas, work, or performance.

This possibility raises an unresolved question: Does fear of negative evaluation simply suppress students’ feedback-seeking, or is it associated with greater use of AI as an alternative feedback source when interpersonal help-seeking is avoided? Existing research on AI feedback has extensively examined feedback generation and delivery, feedback quality, personalized support, and learning outcomes (Venter et al., 2025; Luo et al., 2025; Yildiz Durak and Onan, 2025). However, this literature has largely adopted a feedback-supply perspective, focusing on how AI can generate and deliver effective feedback while paying comparatively limited attention to students’ active feedback-seeking. In particular, little is known about how students’ use of AI as a feedback source relates to their willingness to seek help from interpersonal sources. At the same time, research on feedback-seeking and academic help-seeking has primarily focused on interpersonal sources such as teachers and peers (Leenknecht and Carless, 2023; Joughin et al., 2021), leaving insufficient explanation of how non-interpersonal sources such as AI may reshape students’ feedback-channel choices. More importantly, seeking more AI feedback does not necessarily lead to deeper learning or more substantive revision. Recent evidence suggests that even higher-quality generative AI feedback does not automatically produce better revision outcomes, because students’ interpretation, processing, and uptake of feedback remain critical (Farrokhnia et al., 2026). AI feedback may also introduce suggestions that diverge from students’ existing ideas, and students’ openness to new viewpoints and alternative perspectives may shape whether they examine and use such information in depth (DeYoung et al., 2005; Henderson et al., 2026).

To address these gaps, this study develops an integrated framework linking feedback-source selection with post-feedback learning processing. The framework first examines whether fear of negative evaluation in art learning is associated with AI feedback-seeking through interpersonal help-seeking avoidance, thereby explaining how social-evaluative concerns may reshape rather than simply suppress feedback behavior. It then investigates whether AI feedback-seeking is associated with behavioral engagement in an art-creation task through reflective learning, while testing whether openness affects the extent to which students transform AI-provided information into reflection. AI feedback-seeking serves as the key behavior connecting these two processes. At the front end, it reflects students’ use of AI as a feedback source in relation to interpersonal evaluation concerns; at the back end, it represents the starting point through which students obtain external information and translate it into reflection and action. Accordingly, this study addresses two sequential questions: what interpersonal-evaluative conditions are associated with greater AI feedback-seeking among students engaged in art learning, and how, and under what conditions, such feedback-seeking is associated with positive learning engagement.

This study has some contributions. First, it moves the AI feedback literature beyond the question of whether AI feedback is effective toward an explanation of the conditions associated with students’ active use of AI as a feedback source, thereby incorporating learner agency and feedback-source selection into research on AI-supported learning. Second, it identifies a feedback-source selection pattern, suggesting that fear of negative evaluation may not eliminate the need for feedback but may instead be associated, through interpersonal help-seeking avoidance, with greater use of AI as an alternative feedback source involving less interpersonal exposure. In doing so, the study deepens understanding of evaluation concerns and coping behavior in digital learning environments. Third, it connects feedback-source selection with the subsequent transformation of feedback into learning. By identifying reflective learning as the process linking AI feedback-seeking with task behavioral engagement, and openness as an individual-difference boundary condition, the study develops a process model that integrates social-evaluative concerns, the use of AI feedback resources, and the translation of feedback into learning behavior.

## 2 Theoretical background and hypothesis development

### 2.1 Theoretical foundations

AI feedback-seeking has two theoretical attributes. It may represent a resource-selection response through which students cope with social-evaluative pressure, while also constituting an agentic action through which they actively obtain external information and regulate their learning behavior (Cleavenger and Munyon, 2015; Zhan et al., 2025). The former concerns how individuals appraise interpersonal threats and select coping responses, whereas the latter concerns the interplay among personal factors, environmental resources, and behavioral adjustment. To explain the full process through which AI feedback-seeking emerges and becomes associated with learning consequences, this study integrates transactional stress and coping theory with social cognitive theory.

Transactional stress and coping theory proposes that stress arises from individuals’ cognitive appraisal of the relationship between environmental demands and the coping resources available to them, rather than from the external situation itself (Lazarus and Folkman, 1984). When a situation is appraised as threatening, individuals may adopt cognitive or behavioral strategies to reduce the threat or draw on available resources to manage its demands (Folkman et al., 1986). In art learning, the close connection between artistic work, personal expression, and creative identity may give interpersonal feedback situations heightened social-evaluative significance. The theory provides a basis for understanding why students may avoid feedback channels involving greater interpersonal exposure and turn instead to other available feedback resources. Social cognitive theory, by contrast, emphasizes the reciprocal interplay among personal factors, environmental conditions, and behavior, while viewing learners as agentic actors capable of selecting resources, monitoring their performance, reflecting on their experiences, and adjusting their actions (Bandura, 1989, 2001). From this perspective, AI constitutes an external feedback resource within the learning environment, and students’ active acquisition and use of this resource provide an informational basis for subsequent self-evaluation, reflection, and behavioral adjustment. The two theories offer sequential and complementary explanations within the context of art learning. Transactional stress and coping theory explains how students engaged in creative activities may reconsider feedback sources when interpersonal evaluation threatens their creative self-image, whereas social cognitive theory explains how AI-provided information can be transformed into reflection and subsequent learning behaviors. Thus, AI feedback-seeking is not merely a general information-acquisition behavior; rather, it represents a context-dependent strategy through which art learners manage evaluative concerns and regulate the development of their creative work.

### 2.2 Fear of negative evaluation and interpersonal help-seeking avoidance

Fear of negative evaluation refers to individuals’ concern that others may judge them unfavorably, leading to rejection or damage to their social image (Leary, 1983). In art learning, such concerns may be particularly pronounced because students’ creative works often embody personal ideas, esthetic preferences, and individual styles rather than simply demonstrating task completion. Consequently, negative feedback may be interpreted not only as information about work quality but also as an evaluation of students’ artistic ability, judgment, and creative identity (Fitch, 2023; Halverson et al., 2023).

In art learning, revealing difficulties or presenting underdeveloped work to teachers or peers not only exposes task-related problems but may also be experienced as an evaluation of one’s ability, taste, and creativity. Classroom research has shown that socially evaluative situations, such as responding publicly and being observed by peers, can activate fear of negative evaluation and heighten students’ concerns about making mistakes and exposing limited competence (Downing et al., 2020; Busch et al., 2023). According to transactional stress and coping theory, when an interaction is appraised as threatening to one’s self-image, individuals may manage that threat by reducing contact and limiting exposure (Lazarus and Folkman, 1984; Ryan et al., 2001). Although seeking help can provide information needed to address task difficulties, it may also publicly signal confusion or an inability to complete the task independently. Students who are more sensitive to negative evaluation are therefore more likely to reduce social-evaluative risk by refraining from asking questions or avoiding help-seeking from others (Ryan et al., 2001; Downing et al., 2020). Accordingly, we propose:

*H1*: Fear of negative evaluation in art learning is positively associated with interpersonal help-seeking avoidance.

### 2.3 Interpersonal help-seeking avoidance and AI feedback-seeking

Interpersonal help-seeking avoidance does not necessarily indicate that the need for feedback has disappeared. Rather, it may reflect students’ avoidance of feedback sources associated with greater social-evaluative risk. Uncertainty and the need for improvement remain inherent in artistic creation, yet seeking help from teachers or peers may expose confusion, limited competence, or underdeveloped ideas, thereby imposing reputational and evaluative costs (Ryan et al., 2001; Ashford et al., 2016). According to transactional stress and coping theory, when an established means of accessing resources is appraised as threatening, individuals may turn to alternative resources that can satisfy the same need while involving less threat (Lazarus and Folkman, 1984). Compared with interpersonal help-seeking, AI feedback enables students to obtain diagnostic information, clarification, and suggestions for improvement immediately and repeatedly, with less direct exposure to evaluation. When the need for feedback persists, students who are more inclined to avoid interpersonal help-seeking may therefore also be more inclined to seek feedback from AI as an alternative source involving less interpersonal exposure (Cleavenger and Munyon, 2015; Hou et al., 2024; Zhan et al., 2025). Accordingly, we propose:

*H2*: Interpersonal help-seeking avoidance in art learning is positively associated with AI feedback-seeking.

Extending this reasoning, fear of negative evaluation heightens students’ sensitivity to the risks of interpersonal judgment, thereby increasing their tendency to avoid seeking help from teachers and peers. This avoidance, in turn, may increase the likelihood that they turn to AI for feedback-related information. Thus, interpersonal help-seeking avoidance serves as the mediating mechanism linking fear of negative evaluation with AI feedback-seeking. Accordingly, we propose:

*H3*: Interpersonal help-seeking avoidance mediates the positive relationship between fear of negative evaluation in art learning and AI feedback-seeking.

### 2.4 AI feedback-seeking and reflective learning

AI feedback-seeking not only provides students with information about their current performance but may also prompt them to reconsider their existing ideas. Within artistic creation, feedback processing involves more than identifying errors or correcting deficiencies; it requires learners to interpret feedback in relation to their creative intentions, esthetic decisions, and possible forms of expression (Pitt and Carless, 2022). Such active engagement with feedback is central to reflective learning, as learners need to evaluate external information, reconsider existing approaches, and determine how improvements can be incorporated into subsequent practice (Boud et al., 2013; Kember et al., 2000). Therefore, AI feedback-seeking may serve as a reflective resource that helps art learners examine and refine their creative choices.

According to social cognitive theory, learners actively select and use environmental resources to monitor their performance, evaluate their actions, and adjust subsequent strategies (Bandura, 1989, 2001). When students ask AI to identify problems in their work, explain the basis of its evaluation, or propose alternative approaches, their existing judgments are brought into comparison with new external information. This comparison provides a cognitive basis for questioning established practices, reinterpreting prior experience, and exploring possible directions for improvement. Reflective learning refers precisely to the process through which learners review and reevaluate their actions to develop more effective approaches for subsequent practice (Kember et al., 2000).

Compared with passively receiving AI-generated suggestions, actively seeking diagnostic information, clarification, and improvement advice requires students to recognize gaps in their own understanding and engage in sustained interaction around specific problems. Such behavior is more likely to trigger deeper consideration of their creative choices and the consequences of those choices (Suriano et al., 2025; Pallant et al., 2026). Existing research further indicates that the educational value of AI feedback depends not simply on its availability, but on whether students critically evaluate, actively integrate, and translate it into subsequent action (Helal et al., 2025; Zhan et al., 2025). As students engage in more AI feedback-seeking, they are more likely to compare their original approaches with external suggestions, reevaluate their creative experience, and engage in reflective learning (Zhan and Yan, 2026; Lin et al., 2025; Yuan and Hu, 2025). Accordingly, we propose:

*H4*: AI feedback-seeking in art creation is positively associated with reflective learning.

### 2.5 Reflective learning and task behavioral engagement

Reflective learning enables students to reconsider their existing approaches, evaluate their effectiveness, and formulate more appropriate courses of subsequent action (Kember et al., 2000; Yuan and Hu, 2025). According to social cognitive theory, external information influences goal-directed behavior only when it is processed through self-observation, judgment, and behavioral adjustment (Bandura, 1989). In an art-creation task, reflection can help students understand why their work requires revision, which elements should be retained, and what forms of improvement may be appropriate. In doing so, it reduces uncertainty about how to proceed and provides clearer direction for subsequent effort. Task behavioral engagement reflects students’ effort, attention, persistence, and active involvement in a task (Skinner et al., 2009). When reflection enables students to establish clear improvement goals, they are more likely to continue investing cognitive and behavioral resources in refining their work (Awidi and Klutsey, 2025). Greater reflective learning should therefore be associated with stronger behavioral engagement in the art-creation task. Accordingly, we propose:

*H5*: Reflective learning during art creation is positively associated with task behavioral engagement.

Extending this reasoning, the external information obtained through AI feedback-seeking does not automatically translate into task engagement. Students first evaluate and integrate the feedback through reflection and convert it into actionable directions for revision. Reflective learning constitutes the key mediating process through which AI feedback-seeking is associated with task behavioral engagement. Accordingly, we propose:

*H6*: Reflective learning during art creation mediates the relationship between AI feedback-seeking and task behavioral engagement in art-creation tasks.

### 2.6 The moderating role of openness

Not all students interpret and use the AI feedback they actively seek in the same way. Their willingness to consider different viewpoints, explore unfamiliar information, and reconsider existing judgments may shape how deeply they process feedback and whether feedback-seeking is subsequently translated into reflective learning (Marshall et al., 2021; Dawson et al., 2024). Openness is an important personality trait that captures this individual difference. It reflects individuals’ receptiveness to new ideas, novel experiences, and alternative perspectives, as well as their tendency to explore complex or unfamiliar information (Donnellan et al., 2006).

According to social cognitive theory, personal factors influence how learners interpret and use environmental resources (Bandura, 1989). In an art-creation task, AI feedback may include novel suggestions, different esthetic judgments, or information that conflicts with students’ original ideas. Students higher in openness are more likely to view such discrepancies as cognitive resources worth exploring, compare alternative approaches, and reconsider their existing choices. AI feedback-seeking is therefore more likely to be translated into reflective learning among these students. By contrast, students lower in openness may be more inclined to maintain their initial judgments and engage less deeply with unfamiliar or conflicting suggestions, thereby weakening the relationship between AI feedback-seeking and reflective learning (DeYoung et al., 2005; Marshall et al., 2021). Accordingly, we propose:

*H7*: Openness positively moderates the relationship between AI feedback-seeking and reflective learning, such that the relationship is stronger when openness is higher.

Because reflective learning is, in turn, associated with task behavioral engagement, the strengthening effect of openness should extend to the overall indirect relationship. Specifically, when openness is higher, AI feedback-seeking is more likely to be translated into task behavioral engagement through reflective learning; when openness is lower, this indirect relationship should be weaker. Accordingly, we propose:

*H8*: The indirect relationship between AI feedback-seeking and task behavioral engagement through reflective learning is stronger when openness is higher.

Thus, this study proposes an integrated model linking the antecedents and learning consequences of AI feedback-seeking. The model examines how fear of negative evaluation is associated with AI feedback-seeking through interpersonal help-seeking avoidance, and how AI feedback-seeking is further associated with task behavioral engagement through reflective learning, with openness serving as a boundary condition in this latter process. The full conceptual model is presented in Figure 1.

**Figure 1:**
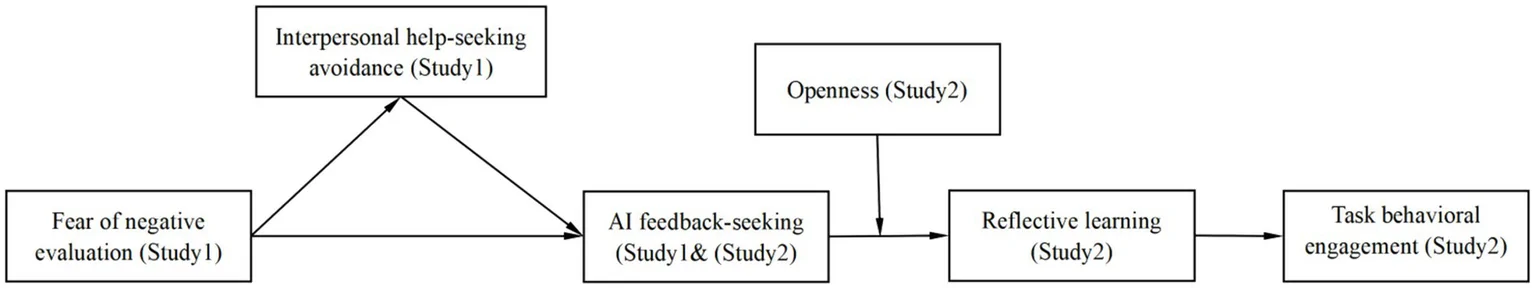
Conceptual model of the antecedents and learning consequences of AI feedback-seeking in art education.

## 3 Overview of the present research

### 3.1 Overall research design

This study employs two complementary studies to test the proposed conceptual model. As shown in Table 1, Study 1 focuses on the psychological antecedents of AI feedback-seeking and uses a survey design to examine the relationships among fear of negative evaluation in art learning, interpersonal help-seeking avoidance, and AI feedback-seeking. Specifically, it investigates whether students’ concerns about interpersonal evaluation are associated with greater avoidance of seeking help from teachers or peers, which in turn is related to a stronger tendency to seek feedback from AI. In doing so, Study 1 examines a feedback-source selection pattern linking interpersonal evaluation concerns, interpersonal help-seeking avoidance, and AI feedback-seeking, and tests H1–H3.

**Table 1:** Overview of the two studies.

Study	Primary objective	Design and context	Main variables	Hypotheses tested
Study 1	To explain why students engaged in art learning seek feedback from AI	Survey of students with art-learning or art-creation experience	Fear of negative evaluation, interpersonal help-seeking avoidance, and AI feedback-seeking	H1–H3
Study 2	To explain how AI feedback-seeking is associated with learning engagement and identify its boundary conditions	Standardized art-creation task in which participants develop an initial proposal, seek AI feedback, and revise their work; task outputs and AI interaction records are retained	AI feedback-seeking, reflective learning, task behavioral engagement, and openness	H4–H8

Study 2 examines the learning consequences of AI feedback-seeking and the boundary conditions of this process. Participants first develop an initial proposal independently in a standardized art-creation task, then use AI to obtain feedback and revise their work. After completing the task, they report their AI feedback-seeking, reflective learning, task behavioral engagement, and openness. Because all participants were required to use the designated AI, AI feedback-seeking in Study 2 captures variation in the extent of feedback inquiry within the task rather than a choice between AI and interpersonal feedback sources. This differs from Study 1, where AI feedback-seeking reflects a general tendency to seek feedback from AI across recent art-learning experiences. Anchoring the Study 2 variables in the same task process, while retaining the initial proposal, revised proposal, and AI interaction records, improves the contextual specificity of the study. Study 2 tests whether AI feedback-seeking is associated with task behavioral engagement through reflective learning and whether openness strengthens this indirect relationship, thereby testing H4–H8. The two studies examine complementary aspects of AI feedback-seeking, but neither directly observes a shift from interpersonal to AI feedback.

### 3.2 Analysis strategy

Partial least squares structural equation modeling (PLS-SEM) was employed to test the proposed hypotheses across the two studies. This approach was appropriate because the conceptual framework involves multiple latent constructs, indirect effects, and a moderated mediation process, while emphasizing explaining variance in the key endogenous variables. The analyses followed the recommended two-stage procedure. First, the measurement models were evaluated in terms of indicator reliability, internal consistency, convergent validity, and discriminant validity. Second, the structural models were assessed by examining path coefficients, explained variance, and predictive relevance.

The significance of the direct and indirect effects was evaluated using bootstrapping with 5,000 resamples and bias-corrected confidence intervals. In Study 1, the analysis tested whether interpersonal help-seeking avoidance mediated the relationship between fear of negative evaluation and AI feedback-seeking. In Study 2, the analysis examined the indirect effect of AI feedback-seeking on task behavioral engagement through reflective learning, the moderating role of openness in the relationship between AI feedback-seeking and reflective learning, and the corresponding conditional indirect effect. The interaction effect between AI feedback-seeking and openness was estimated using the product indicator approach in SmartPLS 4.1.

## 4 Study 1: antecedents of AI feedback-seeking

### 4.1 Methods

#### 4.1.1 Participants and procedure

Participants were recruited through a purposive online sampling procedure targeting higher education students in China with recent experience in art learning and generative AI use. The target population consisted of university-level learners engaged in art education contexts, including students majoring in art-related disciplines and students who had taken art-related courses or participated in art-related creative activities. Because recruitment was conducted through multiple university-related channels, the sample was not restricted to a single institution or geographic area. The survey link was distributed through art-related courses, university student networks, instructors, and online student communities. Recruitment messages invited students to participate in a study on evaluation, help-seeking, and AI use in art learning, without disclosing the hypothesized relationships among the variables.

To ensure alignment between the sample and the research context, eligible participants were required to be at least 18 years old, currently enrolled in higher education, and either studying an art-related major or regularly taking art-related courses during the preceding 12 months. They were also required to have used generative AI at least once for art learning or creation during the preceding 6 months. These criteria ensured that responses reflected recent experience with both interpersonal evaluation in art education and AI-supported learning rather than hypothetical judgments.

Data were collected through Wenjuanxing, an online survey platform widely used in China, between June and July 2025. After providing informed consent and completing the eligibility screening, participants were instructed to answer with reference to their art-learning and creative experiences during the preceding 6 months. The questionnaire took approximately 10–12 min to complete, and participation was voluntary and anonymous. A total of 403 responses were received. After excluding 88 responses that did not meet the prespecified eligibility or response-quality criteria, the final analytical sample comprised 315 participants. Of these, 199 (63.2%) were female and 116 (36.8%) were male. Participants ranged in age from 18 to 28 years, with a mean age of 21.65 years (*SD* = 2.22). Regarding level of study, 99 participants (31.4%) were enrolled in associate or vocational programs, 161 (51.1%) were undergraduate students, and 55 (17.5%) were postgraduate students. In addition, 192 participants (61.0%) were enrolled in art-related majors, while the remaining participants had regularly taken art-related courses.

#### 4.1.2 Measures

All constructs were measured using established multi-item scales adapted to the context of art learning and AI-mediated feedback. The questionnaire was administered in Chinese. The original English items were translated into Chinese and then checked against the source versions to ensure conceptual and semantic equivalence. Discrepancies in wording were reviewed and resolved by the research team before the final questionnaire was administered. A construct-preserving adaptation strategy was adopted: the number of items and the core meaning of each original measure were retained, while referents and situational cues were modified to reflect artistic performance, creative tasks, interpersonal help-seeking, and feedback interactions with generative AI. Participants responded with reference to their experiences in art learning and creation during the preceding 6 months. Unless otherwise specified, all items were rated on a 7-point scale.

Fear of negative evaluation in art learning. This construct was measured using the 12-item brief Fear of Negative Evaluation Scale developed by Leary (1983). Items were contextualized to capture concerns about how teachers, classmates, and others might evaluate participants’ artistic performance, learning, or creative work. A sample item was, “I am frequently afraid that others will notice shortcomings in my art learning, performance, or work.” Interpersonal help-seeking avoidance. Nine items adapted from Pajares et al. (2004) assessed participants’ tendency to avoid seeking assistance from teachers, classmates, or other learners during art courses and creative tasks. A sample item was, “Even if an art-learning or creative task is too difficult to complete on my own, I would not ask others for help.” AI feedback-seeking. Six items adapted from the feedback inquiry measure of Papi et al. (2020) assessed the extent to which participants actively asked generative AI to clarify feedback, identify problems or weaknesses, and provide concrete suggestions for improving their artistic ideas, learning, or work. A sample item was, “I ask the AI to provide specific strategies for improving my art learning, creative idea, or work.”

Marker variable and control variables. To assess potential common method bias (CMB), 4 items measuring general attitudes toward the color blue were included as a theoretically unrelated marker variable and were not incorporated into the substantive model (Miller et al., 2024). Gender, age, and art-related major were included as control variables in the structural model. Gender and art-related major were dummy-coded, whereas age was treated as a continuous variable.

### 4.2 Results

#### 4.2.1 CMB assessment

Because the focal variables in Study 1 were collected from the same respondents using a single survey, the possibility of CMB was examined. An unrotated exploratory factor analysis showed that the first factor accounted for 39.2% of the total variance, indicating that the covariance among the measures was not dominated by a single factor (Podsakoff et al., 2003). A marker-variable analysis provided further evidence (Rönkkö and Ylitalo, 2011; Simmering et al., 2015). The results showed that the marker variable was not significantly associated with either AI feedback-seeking (*β* = −0.089, *p* = 0.422) or interpersonal help-seeking avoidance (*β* = −0.029, *p* = 0.720). Its inclusion also produced only trivial increases in explained variance, from 0.209 to 0.217 for AI feedback-seeking (*ΔR^2^* = 0.008) and from 0.231 to 0.232 for interpersonal help-seeking avoidance (*ΔR^2^* = 0.001). These results suggest that CMB was unlikely to have materially influenced the relationships examined in Study 1.

#### 4.2.2 Measurement model

The measurement model demonstrated satisfactory reliability and validity (Henseler et al., 2015; Hair et al., 2019). All item loadings ranged from 0.703 to 0.896. Cronbach’s α values ranged from 0.901 to 0.944, composite reliability values from 0.924 to 0.953, and average variance extracted (AVE) values from 0.578 to 0.691, supporting internal consistency and convergent validity. Discriminant validity was also established, as the square root of each construct’s AVE exceeded its inter-construct correlations, while all HTMT values ranged from 0.259 to 0.491, below the recommended threshold of 0.85.

#### 4.2.3 Structural model and hypothesis testing

The structural model was assessed using PLS-SEM, with the significance of the hypothesized relationships evaluated through bias-corrected bootstrapping with 5,000 resamples. As shown in Table 2, the model showed a satisfactory level of model fit, with an SRMR value of 0.055. The coefficient of determination (*R^2^*) indicated that the model explained 23.1% of the variance in interpersonal help-seeking avoidance and 20.9% of the variance in AI feedback-seeking. Although the explained variance was relatively modest, this result is theoretically acceptable because Study 1 focuses on a specific psychological pathway through which evaluation concerns and interpersonal help-seeking avoidance are associated with AI feedback-seeking, rather than aiming to capture all potential psychological and contextual factors influencing students’ use of AI as a feedback source. The corresponding *Q^2^* values were 0.222 and 0.040, respectively, indicating predictive relevance for both endogenous constructs.

**Table 2:** Structural model assessment (Study 1).

Construct	*R* ^2^	*Q* ^2^	SRMR
AIFS	0.209	0.040	0.055
IHA	0.231	0.222	

AIFS, AI feedback-seeking; IHA, Interpersonal help-seeking avoidance.

As shown in Table 3, fear of negative evaluation was positively associated with interpersonal help-seeking avoidance (*β* = 0.480, 95% CI [0.403, 0.565], *p* < 0.001), supporting H1. Interpersonal help-seeking avoidance was subsequently associated with greater AI feedback-seeking (*β* = 0.422, 95% CI [0.299, 0.539], *p* < 0.001), supporting H2.

**Table 3:** The results of hypothesis testing (Study 1).

Effect	*β*-value	LLCI	ULCI	*p*-value	Role	Support
Panel A. Hypothesized structural relationships						
FNE → IHA	0.480	0.403	0.565	<0.001	H1	Yes
IHA → AIFS	0.422	0.299	0.539	<0.001	H2	Yes
Panel B. Main effect and mediation decomposition						
FNE → AIFS (total effect)	0.254	0.156	0.359	<0.001	H3 decomposition	—
FNE → AIFS (direct effect)	0.052	−0.066	0.173	0.404	H3 decomposition	—
FNE → IHA → AIFS (indirect effect)	0.203	0.139	0.277	<0.001	H3	Yes
Panel C. Control variables						
Gender → AIFS	−0.030	−0.238	0.180	0.776	Control	—
Age → AIFS	0.025	−0.069	0.117	0.591	Control	—
Art-related major → AIFS	−0.136	−0.340	0.063	0.190	Control	—

LLCI, lower limit of the 95% bootstrap confidence interval; ULCI, upper limit of the 95% bootstrap confidence interval; FNE, fear of negative evaluation; IHA, interpersonal help-seeking avoidance; AIFS, AI feedback-seeking.

The mediation analysis further showed that interpersonal help-seeking avoidance significantly mediated the relationship between fear of negative evaluation and AI feedback-seeking (*β* = 0.203, 95% CI [0.139, 0.277], *p* < 0.001), supporting H3. The total effect of fear of negative evaluation on AI feedback-seeking was significant (*β* = 0.254, 95% CI [0.156, 0.359], *p* < 0.001), whereas the direct effect became nonsignificant after including the mediator (*β* = 0.052, 95% CI [−0.066, 0.173], *p* = 0.404). These results indicate that interpersonal help-seeking avoidance serves as the primary mechanism linking fear of negative evaluation with AI feedback-seeking. Finally, the control variables, including gender, age, and art-related major, were not significantly related to AI feedback-seeking.

## 5 Study 2: consequences of AI feedback-seeking in a standardized art task

### 5.1 Methods

#### 5.1.1 Participants and procedure

Participants were recruited from art-related programs and courses at two undergraduate institutions in Zhejiang Province, China. The target population consisted of higher education students in art education contexts who were enrolled in art-related majors or had relevant experience through art-related coursework. A total of 200 students were initially recruited, with 100 students recruited from each institution. Eligible participants were required to be at least 18 years old, to be enrolled in an art-related major or to have regularly taken art-related courses during the preceding 12 months, and to be able to use the designated generative AI tool. The study was completed in a single standardized session. After providing informed consent, participants completed a pre-task questionnaire, the standardized art-creation task, and a post-task questionnaire. Participation was voluntary, and platform-generated anonymous identifiers were used to link questionnaire responses, task outputs, and AI interaction records without collecting names, student numbers, or other directly identifying information. A total of 28 cases were excluded because they did not meet the prespecified procedural or data-quality criteria, resulting in a final analytical sample of 172 participants. The final sample included 98 female participants (57.0%) and 74 male participants (43.0%). The mean age of the participants was 20.18 years (*SD* = 1.92).

#### 5.1.2 Standardized art-creation task

Participants completed a standardized creative-planning task that required them to develop a proposal for a campus art experience activity around the theme “Connection.” The activity was intended to help university students experience art, express ideas, and interact with others through an accessible artistic form. Participants could use music, visual art, photography, performance, film, crafts, installation, or a combination of media. Each proposal was required to include an activity title, the principal artistic form, the central creative idea, the main participant activity, and an explanation of how the proposal represented the theme. The required length was 200–300 Chinese characters or 150–200 English words, and no drawing, poster production, or specialist design software was required.

The task consisted of two consecutive stages. Participants first had 10 min to produce and submit an initial proposal independently, without using generative AI, search engines, online materials, or assistance from others. This stage ensured that the subsequent AI interaction was based on a participant-generated idea rather than on asking AI to create a complete proposal from scratch. Participants were then given 15 min to refine their proposal using the same designated generative AI tool. They were free to decide how to interact with the AI, including requesting evaluation, clarification, problem diagnosis, alternative perspectives, or concrete suggestions for improvement, and no prompt template or sample response was provided. The theme, instructions, time allocation, AI tool, and required outputs were held constant across participants, while the extent and form of AI feedback-seeking were allowed to vary naturally. The initial proposal, revised proposal, and complete AI interaction record were retained to verify task completion.

#### 5.1.3 Measures

All focal constructs were measured using established multi-item scales adapted to the standardized art-creation task. The questionnaire was administered in Chinese, following the same translation and wording-verification procedure described in Study 1. Openness was assessed before the task, whereas AI feedback-seeking, reflective learning, and task behavioral engagement were measured immediately after the AI-assisted refinement stage. The number of items and the core meaning of the original measures were retained, with the referents, situational cues, and tense adjusted where necessary to capture participants’ actual task experience. Unless otherwise indicated, all items were rated on a 7-point scale. Gender, age, and AI tool familiarity were included as control variables in the structural model. Gender was dummy-coded, age was treated as a continuous variable, and AI tool familiarity was measured using a single-item measure asking participants, “How familiar are you with generative AI tools?” Responses were recorded on a 7-point scale ranging from 1 (not at all familiar) to 7 (extremely familiar).

Openness was measured using the 4-item openness subscale of the Mini-IPIP developed by Donnellan et al. (2006). A sample item was, “I have a vivid imagination.” AI feedback-seeking was assessed after the task using the same six-item measure as in Study 1. The wording was adjusted only to refer to participants’ interaction with the designated AI during the art-creation task and to their creative idea or proposal. The items assessed the extent to which participants requested clarification, problem diagnosis, identification of weaknesses, and concrete improvement strategies. A sample item was, “I asked the AI to provide specific strategies for improving my creative idea or proposal.” Reflective learning was measured using 4 items adapted from Kember et al. (2000). The items assessed the extent to which participants reconsidered their initial ideas, questioned existing approaches, and explored alternative ways of completing the task. A sample item was, “I thought over what I had been doing and considered alternative ways of doing it.” Task behavioral engagement was assessed using 5 items adapted from Skinner et al. (2009). The items captured participants’ effort, active involvement, and sustained attention while using AI to refine their proposals. A sample item was, “While using AI to refine the proposal, I worked as hard as I could.”

### 5.2 Results

#### 5.2.1 CMB assessment

Harman’s single-factor test showed that the first unrotated factor explained 32.7% of the total variance, indicating that no single factor dominated the measures (Podsakoff et al., 2003). A latent common method factor analysis was then conducted for further assessment (Liang et al., 2007). Most method-factor loadings were nonsignificant, and the method factor explained only 0.9% of the indicator variance on average, compared with 72.1% explained by the substantive factors. These results indicate that CMB was unlikely to materially affect the findings.

#### 5.2.2 Measurement model

The measurement model showed satisfactory reliability and validity (Henseler et al., 2015; Hair et al., 2019). Indicator loadings ranged from 0.712 to 0.948. Cronbach’s α values ranged from 0.888 to 0.922, composite reliability values from 0.918 to 0.940, and AVE values from 0.652 to 0.796, supporting internal consistency and convergent validity. Discriminant validity was also established, as the square root of each construct’s AVE exceeded its correlations with the other constructs, and all HTMT ratios ranged from 0.118 to 0.522.

#### 5.2.3 Structural model and hypothesis testing

The structural model was assessed using PLS-SEM. The significance of the path coefficients and indirect relationships was examined through bias-corrected bootstrapping with 5,000 resamples. As shown in Table 4, the model demonstrated a satisfactory level of model fit, with an SRMR value of 0.058. The *R^2^* indicated that the model explained 36.7% of the variance in reflective learning and 25.1% of the variance in task behavioral engagement. These results suggest moderate explanatory power for the proposed learning-process model, which focuses on how AI feedback-seeking is transformed into reflective learning and subsequent behavioral engagement rather than attempting to explain all possible determinants of students’ learning behaviors. The corresponding *Q^2^* values were 0.339 and 0.106, respectively, indicating predictive relevance for both endogenous constructs.

**Table 4:** Structural model assessment (Study 2).

Construct	*R^2^*	*Q^2^*	SRMR
RL	0.367	0.339	0.058
TBE	0.251	0.106	

RL, reflective learning; TBE, task behavioral engagement.

As shown in Table 5, AI feedback-seeking was positively associated with reflective learning (*β* = 0.500, 95% confidence interval [0.380, 0.613], *p* < 0.001), supporting H4. Reflective learning was, in turn, positively associated with task behavioral engagement (*β* = 0.465, 95% confidence interval [0.319, 0.616], *p* < 0.001), supporting H5. The total effect of AI feedback-seeking on task behavioral engagement was significant (*β* = 0.250, 95% confidence interval [0.121, 0.379], *p* < 0.001), whereas the direct effect was not significant after reflective learning was included in the model (*β* = 0.018, 95% confidence interval [−0.137, 0.168], *p* = 0.821). More importantly, the indirect effect of AI feedback-seeking on task behavioral engagement through reflective learning was significant (*β* = 0.232, 95% confidence interval [0.141, 0.341], *p* < 0.001), supporting H6. These results indicate that the positive association between AI feedback-seeking and task behavioral engagement was primarily transmitted through reflective learning.

**Table 5:** The results of hypothesis testing (Study 2).

Effect	*β*-value	LLCI	ULCI	*p*-value	Role	Support
Panel A. Hypothesized structural relationships						
AIFS → RL	0.500	0.380	0.613	<0.001	H4	Yes
RL → TBE	0.465	0.319	0.616	<0.001	H5	Yes
OPEN × AIFS → RL	0.317	0.115	0.429	<0.001	H7	Yes
Index of moderated mediation	0.147	0.049	0.220	0.001	H8	Yes
Panel B. Main effect and mediation decomposition						
OPEN → RL	0.152	−0.100	0.281	0.080	Moderator main effect	—
AIFS → TBE (total effect)	0.250	0.121	0.379	<0.001	H6 decomposition	—
AIFS → TBE (direct effect)	0.018	−0.137	0.168	0.821	H6 decomposition	—
AIFS → RL → TBE (indirect effect)	0.232	0.141	0.341	<0.001	H6	Yes
Panel C. Control variables						
Age → TBE	0.115	−0.021	0.257	0.106	Control	—
Gender → TBE	0.190	−0.076	0.470	0.172	Control	—
AI tool familiarity → TBE	−0.064	−0.190	0.064	0.323	Control	—

LLCI, lower limit of the 95% bootstrap confidence interval; ULCI, upper limit of the 95% bootstrap confidence interval; AIFS, AI feedback-seeking; OPEN, openness; RL, reflective learning; TBE, task behavioral engagement.

Beyond the mediation mechanism, the moderating role of openness was further examined. Openness significantly moderated the positive relationship between AI feedback-seeking and reflective learning (*β* = 0.317, 95% confidence interval [0.115, 0.429], *p* < 0.001), supporting H7. As shown in Figure 2, the simple-slope analysis indicated that the positive relationship between AI feedback-seeking and reflective learning was weaker at low levels of openness (−1 *SD*) and stronger at high levels of openness (+1 *SD*). In addition, the index of moderated mediation was significant (*β* = 0.147, 95% confidence interval [0.049, 0.220], *p* = 0.001), supporting H8. Figure 3 further showed that the indirect effect of AI feedback-seeking on task behavioral engagement through reflective learning increased from low openness (−1 *SD*) to high openness (+1 *SD*), indicating that openness strengthened the indirect relationship.

**Figure 2:**
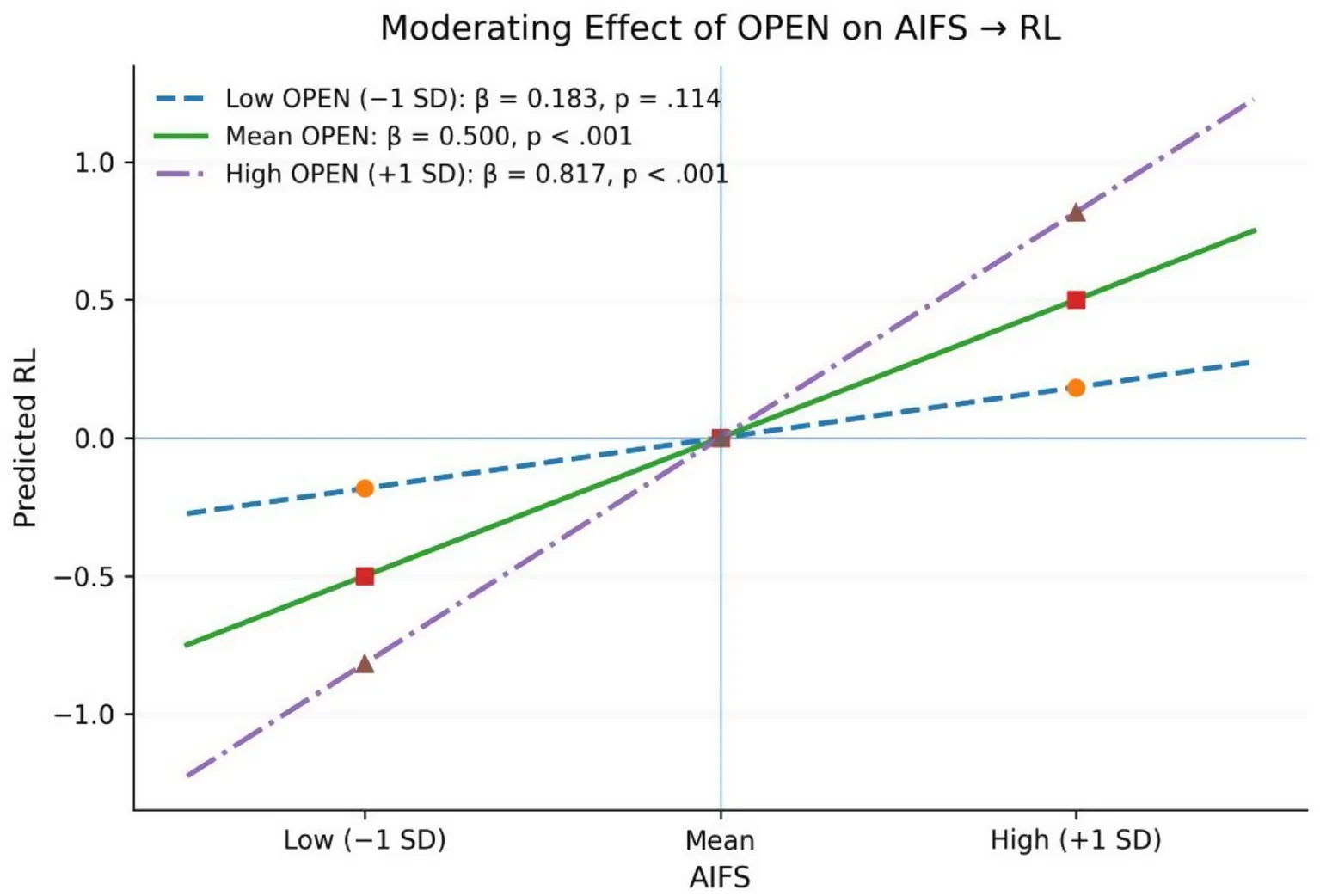
Simple slopes of AIFS predicting RL at different levels of OPEN.

**Figure 3:**
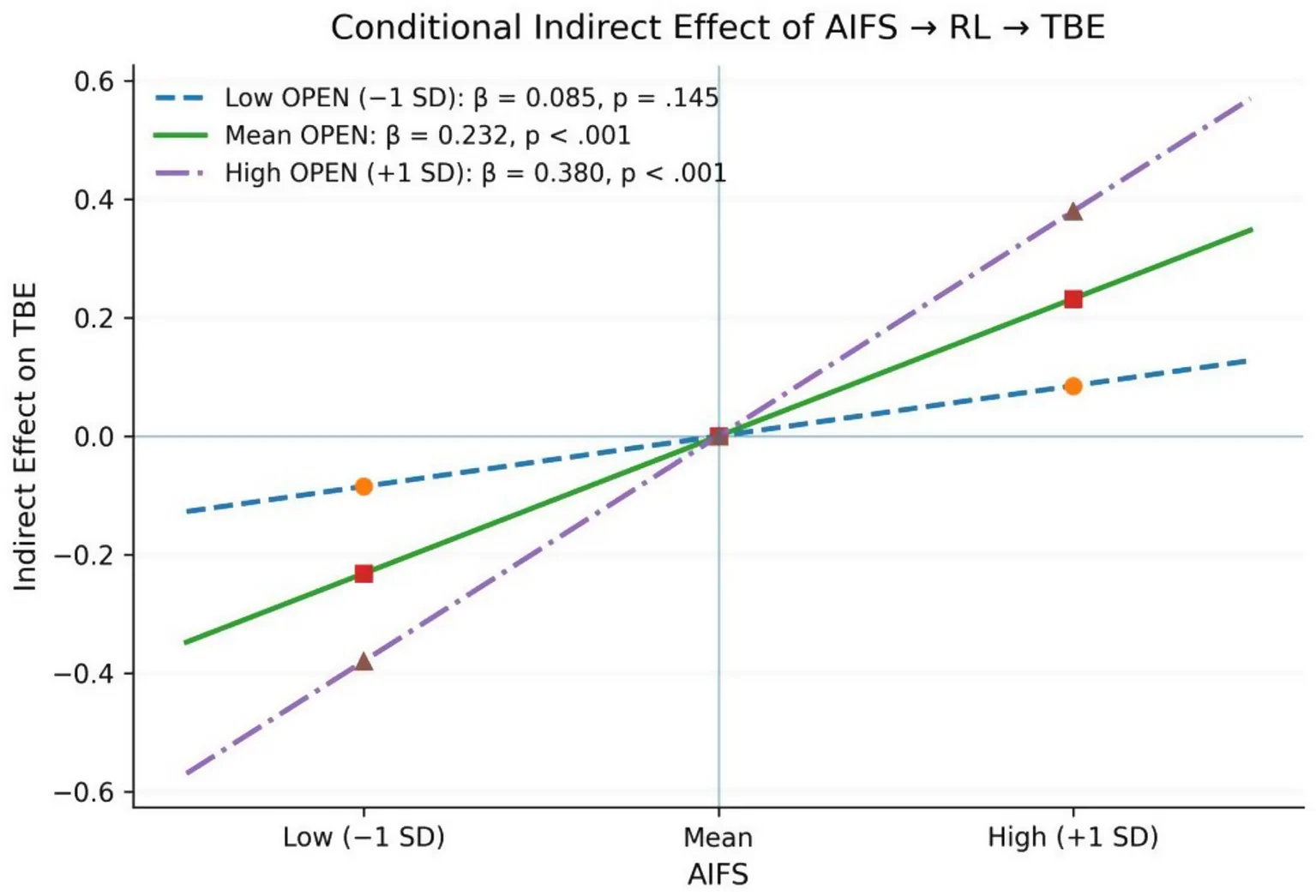
Simple slopes of the indirect effect of AIFS on TBE via RL at different levels of OPEN.

## 6 General discussion

### 6.1 Summary of findings

Drawing on two complementary studies, this research examined why students seek feedback from AI and how such feedback-seeking is associated with engagement in an art-creation task. Study 1 showed that fear of negative evaluation was positively associated with interpersonal help-seeking avoidance and, indirectly, with greater AI feedback-seeking. Study 2 showed that AI feedback-seeking was associated with task behavioral engagement through reflective learning, with this process becoming stronger at higher levels of openness.

The findings of Study 1 reveal a selective pattern of feedback-seeking under conditions of evaluation concern. Fear of negative evaluation was associated with greater avoidance of seeking help from teachers or peers, which is consistent with prior work showing that students may refrain from requesting assistance when doing so exposes uncertainty or threatens perceptions of competence (Ryan et al., 2001; Downing et al., 2020). Interpersonal help-seeking avoidance was nevertheless associated with greater AI feedback-seeking. Thus, reluctance to approach human sources did not coincide with a general withdrawal from feedback. Instead, students who were more inclined to avoid interpersonal help were also more likely to seek diagnostic and improvement-oriented information from AI (Reinhardt et al., 2025). This result accords with evidence that computer-mediated channels can facilitate help-seeking by changing the self-presentational conditions surrounding the request for assistance (Cleavenger and Munyon, 2015). In the context of generative AI, students may therefore distinguish between the need for feedback and the interpersonal exposure involved in obtaining it.

Study 2 extends this pattern by showing that the relevance of AI feedback-seeking to task engagement depended on reflective learning. Students who sought more evaluation, clarification, and improvement suggestions from AI also reported greater reconsideration of their initial ideas and exploration of alternative approaches (Yuan and Hu, 2025). Reflective learning was, in turn, associated with stronger behavioral engagement in the task. The relatively stronger association between AI feedback-seeking and reflective learning than the direct association between AI feedback-seeking and task behavioral engagement further suggests that AI feedback-seeking contributes to learning primarily by initiating reflective processing rather than by directly producing behavioral engagement. This finding is consistent with the view that students are not passive recipients of AI-generated feedback; the educational value of such feedback depends on how actively they interpret, evaluate, and use it (Zhan et al., 2025). Empirical work on students’ engagement with ChatGPT feedback similarly shows that learners vary in the extent to which they interrogate, elaborate on, or simply accept the information provided (Zhan and Yan, 2026). Accordingly, the non-significant direct effect of AI feedback-seeking on task behavioral engagement after reflective learning was included indicates that obtaining AI feedback alone may not be sufficient to enhance engagement unless students actively interpret, evaluate, and integrate the feedback into their creative process. The present results suggest that reflective processing is the point at which AI feedback-seeking becomes meaningfully connected with sustained effort and involvement in the creative task.

Openness further qualified this process. The association between AI feedback-seeking and reflective learning was stronger among students higher in openness, and the corresponding indirect association with task behavioral engagement followed the same pattern. AI feedback may introduce unfamiliar suggestions, alternative interpretations, or ideas that depart from a student’s original creative direction. Because openness reflects a tendency toward cognitive exploration and flexibility when engaging with novel information, students higher in openness may be more willing to examine such discrepancies and reconsider their initial judgments (DeYoung et al., 2005). Students lower in openness, by contrast, may seek feedback without engaging with divergent suggestions to the same extent. The findings therefore indicate that AI feedback-seeking is not uniformly associated with deeper task engagement; its relevance depends on both reflective processing and learners’ receptiveness to perspectives beyond their initial approach.

### 6.2 Theoretical contributions

The study’s primary theoretical contribution lies in distinguishing avoidance of interpersonal exposure from withdrawal from feedback. Transactional stress and coping theory explains how individuals respond to demands by appraising both the threat involved and the resources available to manage it (Lazarus and Folkman, 1984; Folkman et al., 1986). In AI-supported learning environments, however, feedback is no longer tied exclusively to interpersonal exchange. Students may remain motivated to obtain evaluative information while differing in the source through which they access it. This distinction extends the application of stress and coping theory beyond a simple approach–avoidance account by suggesting that responses to evaluation concerns may also involve selective access to resources that differ in social exposure. This interpretation concerns feedback-source selection rather than directly observed substitution: in Study 1, AI feedback-seeking reflected a general tendency associated with interpersonal help-seeking avoidance, whereas in Study 2 it reflected variation in feedback-seeking intensity within a task in which all participants used AI. Accordingly, neither study directly observed a shift from interpersonal to AI feedback.

AI feedback-seeking consequently occupies a theoretically distinctive position at the intersection of coping and agency. It may be associated with students’ management of interpersonal evaluation concerns, yet it also represents the deliberate selection and use of an environmental resource. Social cognitive theory emphasizes that learners exercise agency by seeking information, monitoring their performance, and adjusting their subsequent actions (Bandura, 1989, 2001). Viewed in this way, AI feedback-seeking should not be treated as merely a defensive retreat from human interaction or as inherently productive technology use. Its theoretical meaning depends on both the conditions associated with the use of different feedback sources and the way the acquired information is subsequently used.

The integration of transactional stress and coping theory with social cognitive theory further provides a process account that connects feedback-resource selection with learning regulation. The former helps explain why students may differ in where they seek feedback under evaluation concerns, whereas the latter clarifies how information obtained from AI may become meaningfully associated with subsequent engagement through reflective processing. Openness adds an important boundary to this process by indicating that the relationship is stronger among learners who are more receptive to unfamiliar ideas and alternative perspectives (DeYoung et al., 2005). The resulting framework avoids assuming that access to AI feedback is uniformly beneficial. Instead, it locates the learning relevance of AI feedback-seeking in the interplay among source selection, reflective processing, and learners’ readiness to reconsider their initial approach.

### 6.3 Practical implications

For art educators, AI feedback should be positioned as a complement to, rather than a competitor of, teacher and peer feedback. In particular, a low-threat AI pre-feedback stage can be introduced before classroom critique or individual consultation. Students who feel uncomfortable exposing unfinished ideas or uncertainties to others may first use AI to clarify their concerns, identify aspects of the work that require attention, and organize the questions they wish to discuss. Teacher feedback can then address issues that require contextual understanding, disciplinary expertise, and sensitivity to the student’s creative intention. Such an arrangement preserves the distinctive value of interpersonal feedback while offering students a more gradual entry into evaluative dialog.

The effectiveness of this arrangement also depends on how students use AI. AI should be approached primarily as a resource for diagnosing problems, clarifying feedback, and comparing possible directions, rather than as a tool for directly generating or completing creative work. Students need to examine whether AI suggestions are consistent with the purpose, meaning, and esthetic choices of their work, particularly when the advice appears persuasive but conflicts with their original intention. They should therefore be encouraged to question, compare, selectively adopt, or reject AI suggestions instead of treating them as authoritative judgments. The aim is not simply to obtain more feedback, but to use feedback in ways that preserve critical judgment and creative ownership.

Course and platform design should, in turn, support these forms of feedback use. AI-supported activities can be structured around follow-up questioning, explanation, comparison, and reflection, enabling students to move beyond receiving a single answer and to examine the reasoning and alternatives behind the feedback. Platforms can provide prompts that invite students to request clarification, compare competing suggestions, and explain their eventual choices. By contrast, functions that offer only one-way recommendations or directly replace students’ task performance should play a limited role in feedback-oriented activities. Designing AI around dialog and deliberation, rather than automatic completion, can better support its integration into the reflective and interpretive practices of art education.

### 6.4 Limitations and future research

Several limitations should be acknowledged. First, the findings should be interpreted with caution regarding causal inference. Although Study 2 adopted a standardized art-creation task, the focal variables were measured through post-task self-reports, and the cross-sectional nature of the data does not allow definitive conclusions about temporal relationships. Moreover, unmeasured individual differences, such as task motivation or conscientiousness, may have simultaneously influenced students’ AI feedback-seeking, reflective learning, and task engagement. Future research could address these issues by incorporating relevant covariates and using longitudinal, experimental, or behavioral approaches to further validate the proposed relationships. Second, the generalizability of the findings is constrained by the research context. The samples were drawn from students with art-learning experience in China, and Study 2 involved a single standardized creative task and a designated AI tool. Future research should examine whether the proposed model extends across different cultural settings, educational contexts, artistic disciplines, task types, and AI systems. Third, this study examined openness as one boundary condition influencing how AI feedback-seeking is translated into reflective learning. However, other individual and situational factors, such as feedback literacy, prior AI experience, creative self-efficacy, and perceived AI feedback quality, may also shape this process. Future studies could extend the current framework by exploring additional mechanisms and boundary conditions underlying students’ interpretation and use of AI-generated feedback.

## Data Availability

The raw data supporting the conclusions of this article will be made available by the authors, without undue reservation.

## References

[ref1] Ashford S. J. De Stobbeleir K. Nujella M. (2016). To seek or not to seek: is that the only question? Recent developments in feedback-seeking literature. Annu. Rev. Organ. Psychol. Organ. Behav. 3, 213–239. doi: 10.1146/annurev-orgpsych-041015-062314

[ref2] Awidi I. T. Klutsey J. Q. (2025). Using online critical reflection to enhance students’ confidence, motivation, and engagement in higher education. Technol. Knowl. Learn. 30, 1499–1534. doi: 10.1007/s10758-024-09751-4

[ref3] Bandura A. (1989). Human agency in social cognitive theory. Am. Psychol. 44, 1175–1184. doi: 10.1037/0003-066X.44.9.1175, 2782727

[ref4] Bandura A. (2001). Social cognitive theory: an agentic perspective. Annu. Rev. Psychol. 52, 1–26. doi: 10.1146/annurev.psych.52.1.1, 11148297

[ref5] eds. Boud D. Keogh R. Walker D. (2013). Reflection: Turning Experience Into Learning. London: Routledge.

[ref6] Busch C. A. Wiesenthal N. J. Mohammed T. F. Anderson S. Barstow M. Custalow C. . (2023). The disproportionate impact of fear of negative evaluation on first-generation college students, LGBTQ+ students, and students with disabilities in college science courses. CBE—Life Sci. Educ. 22:ar31. doi: 10.1187/cbe.22-10-0195 PMC10424224 37347813

[ref7] Cleary Rodrigues M. C. (2025). The crit: making meaning with peers in fine art studio pedagogy. Arts Humanit. High. Educ. 24, 278–291. doi: 10.1177/14740222241311857

[ref8] Cleavenger D. J. Munyon T. P. (2015). Overcoming the help-seeker’s dilemma: how computer-mediated systems encourage employee help-seeking initiation. Organ. Stud. 36, 221–240. doi: 10.1177/0170840614556920

[ref9] Dawson P. Yan Z. Lipnevich A. Tai J. Boud D. Mahoney P. (2024). Measuring what learners do in feedback: the feedback literacy behaviour scale. Assess. Eval. High. Educ. 49, 348–362. doi: 10.1080/02602938.2023.2240983

[ref10] DeYoung C. G. Peterson J. B. Higgins D. M. (2005). Sources of openness/intellect: cognitive and neuropsychological correlates of the fifth factor of personality. J. Pers. 73, 825–858. doi: 10.1111/j.1467-6494.2005.00330.x, 15958136

[ref11] Donnellan M. B. Oswald F. L. Baird B. M. Lucas R. E. (2006). The mini-IPIP scales: tiny-yet-effective measures of the big five factors of personality. Psychol. Assess. 18, 192–203. doi: 10.1037/1040-3590.18.2.192, 16768595

[ref12] Downing V. R. Cooper K. M. Cala J. M. Gin L. E. Brownell S. E. (2020). Fear of negative evaluation and student anxiety in community college active-learning science courses. CBE Life Sci. Educ. 19:ar20. doi: 10.1187/cbe.19-09-0186, 32453679 PMC8697658

[ref13] Farrokhnia M. Latifi S. Papadopoulos P. M. Hogenkamp L. Gijlers H. Khosravi H. . (2026). Generative AI offers more, but students revise less: comparing the effects of teacher and AI feedback on student essay revisions. Int. J. Educ. Technol. High. Educ. 23:6. doi: 10.1186/s41239-026-00579-9

[ref14] Fitch S. (2023). Art, assessment and uncertainty. Art Des. Commun. High. Educ. 22, 263–275. doi: 10.1386/adch_00077_1

[ref15] Folkman S. Lazarus R. S. Dunkel-Schetter C. DeLongis A. Gruen R. J. (1986). Dynamics of a stressful encounter: cognitive appraisal, coping, and encounter outcomes. J. Pers. Soc. Psychol. 50, 992–1003. doi: 10.1037/0022-3514.50.5.992, 3712234

[ref16] Hair J. F. Risher J. J. Sarstedt M. Ringle C. M. (2019). When to use and how to report the results of PLS-SEM. Eur. Bus. Rev. 31, 2–24. doi: 10.1108/EBR-11-2018-0203

[ref17] Halverson E. R. Saplan K. Mejias S. Martin C. (2023). What we learn about learning from out-of-school time arts education. Rev. Res. Educ. 47, 360–404. doi: 10.3102/0091732X231223492

[ref18] Helal M. Y. Elgendy I. A. Albashrawi M. A. Dwivedi Y. K. Al-Ahmadi M. S. Jeon I. (2025). The impact of generative AI on critical thinking skills: a systematic review, conceptual framework and future research directions. Inf. Discov. Delivery. doi: 10.1108/IDD-05-2025-0125

[ref19] Henderson M. Bearman M. Chung J. Fawns T. Buckingham Shum S. Matthews K. E. . (2026). Comparing generative AI and teacher feedback: student perceptions of usefulness and trustworthiness. Assess. Eval. High. Educ., 51: 863–878. doi: 10.1080/02602938.2025.2502582

[ref20] Henseler J. Ringle C. M. Sarstedt M. (2015). A new criterion for assessing discriminant validity in variance-based structural equation modeling. J. Acad. Mark. Sci. 43, 115–135. doi: 10.1007/s11747-014-0403-8

[ref21] Hou I. Mettille S. Man O. Li Z. Zastudil C. MacNeil S. (2024). “The effects of generative AI on computing students’ help-seeking preferences,” in Proceedings of the 26th Australasian Computing Education Conference, (New York, NY: Association for Computing Machinery (ACM)), 39–48.

[ref22] Joughin G. Boud D. Dawson P. Tai J. (2021). What can higher education learn from feedback seeking behaviour in organisations? Implications for feedback literacy. Assess. Eval. High. Educ. 46, 80–91. doi: 10.1080/02602938.2020.1733491

[ref23] Kember D. Leung D. Y. Jones A. Loke A. Y. McKay J. Sinclair K. . (2000). Development of a questionnaire to measure the level of reflective thinking. Assess. Eval. High. Educ. 25, 381–395. doi: 10.1080/713611442

[ref24] Lazarus R. S. Folkman S. (1984). Stress, Appraisal, and Coping. New York, NY: Springer Publishing Company.

[ref25] Leary M. R. (1983). A brief version of the fear of negative evaluation scale. Personal. Soc. Psychol. Bull. 9, 371–375. doi: 10.1177/0146167283093007

[ref26] Leenknecht M. J. Carless D. (2023). Students’ feedback seeking behaviour in undergraduate education: a scoping review. Educ. Res. Rev. 40:100549. doi: 10.1016/j.edurev.2023.100549

[ref27] Liang H. Saraf N. Hu Q. Xue Y. (2007). Assimilation of enterprise systems: the effect of institutional pressures and the mediating role of top management. MIS Q. 31, 59–87. doi: 10.2307/25148781

[ref28] Lin C.-J. Lee H.-Y. Wang W.-S. Huang Y.-M. Wu T.-T. (2025). Enhancing reflective thinking in STEM education through experiential learning: the role of generative AI as a learning aid. Educ. Inf. Technol. 30, 6315–6337. doi: 10.1007/s10639-024-13072-5

[ref29] Luo J. Zheng C. Yin J. Teo H. H. (2025). Design and assessment of AI-based learning tools in higher education: a systematic review. Int. J. Educ. Technol. High. Educ. 22:42. doi: 10.1186/s41239-025-00540-2

[ref30] Marshall T. Keville S. Cain A. Adler J. R. (2021). On being open-minded, wholehearted, and responsible: a review and synthesis exploring factors enabling practitioner development in reflective practice. Reflective Pract. 22, 860–876. doi: 10.1080/14623943.2021.1976131

[ref31] Miller B. Simmering M. Ragland E. (2024). Effective and adaptable: four studies on the shortened attitude toward the color blue marker variable scale. Behav. Res. Methods 56, 7985–8008. doi: 10.3758/s13428-024-02465-6, 39009822

[ref32] Pajares F. Cheong Y. F. Oberman P. (2004). Psychometric analysis of computer science help-seeking scales. Educ. Psychol. Meas. 64, 496–513. doi: 10.1177/0013164403258447

[ref33] Pallant J. L. Blijlevens J. Campbell A. Jopp R. (2026). Mastering knowledge: the impact of generative AI on student learning outcomes. Stud. High. Educ. 51, 714–735. doi: 10.1080/03075079.2025.2487570

[ref34] Papi M. Bondarenko A. V. Wawire B. Jiang C. Zhou S. (2020). Feedback-seeking behavior in second language writing: motivational mechanisms. Read. Writ. 33, 485–505. doi: 10.1007/s11145-019-09971-6

[ref35] Pitt E. Carless D. (2022). Signature feedback practices in the creative arts: integrating feedback within the curriculum. Assess. Eval. High. Educ. 47, 817–829. doi: 10.1080/02602938.2021.1980769

[ref36] Podsakoff P. M. MacKenzie S. B. Lee J.-Y. Podsakoff N. P. (2003). Common method biases in behavioral research: a critical review of the literature and recommended remedies. J. Appl. Psychol. 88, 879–903. doi: 10.1037/0021-9010.88.5.879, 14516251

[ref37] Reinhardt A. Matthes J. Bojic L. Maindal H. T. Paraschiv C. Ryom K. (2025). Help me, doctor AI? A cross-national experiment on the effects of disease threat and stigma on AI health information-seeking intentions. Comput. Hum. Behav. 172:108718. doi: 10.1016/j.chb.2025.108718

[ref38] Rönkkö M. Ylitalo J. (2011). “PLS marker variable approach to diagnosing and controlling for method variance,” in Proceedings of the 32nd International Conference on Information Systems (ICIS 2011), (Atlanta, GA: Association for Information Systems).

[ref39] Ryan A. M. Pintrich P. R. Midgley C. (2001). Avoiding seeking help in the classroom: who and why? Educ. Psychol. Rev. 13, 93–114. doi: 10.1023/A:1009013420053

[ref40] Schmitt-Fumian T. K. Tauscher S. Thoring K. (2025). AI vs. human: exploring the potential of generative AI as a feedback tool to support ideation in design education. Proc. Des. Soc. 5, 429–438. doi: 10.1017/pds.2025.10057

[ref41] Simmering M. J. Fuller C. M. Richardson H. A. Ocal Y. Atinc G. M. (2015). Marker variable choice, reporting, and interpretation in the detection of common method variance: a review and demonstration. Organ. Res. Methods 18, 473–511. doi: 10.1177/1094428114560023

[ref42] Skinner E. A. Kindermann T. A. Furrer C. J. (2009). A motivational perspective on engagement and disaffection: conceptualization and assessment of children's behavioral and emotional participation in academic activities in the classroom. Educ. Psychol. Meas. 69, 493–525. doi: 10.1177/0013164408323233

[ref43] Smith C. (2011). Understanding students’ views of the crit assessment. J. Educ. Built Environ. 6, 44–67. doi: 10.11120/jebe.2011.06010044

[ref44] Suriano R. Plebe A. Acciai A. Fabio R. A. (2025). Student interaction with ChatGPT can promote complex critical thinking skills. Learn. Instr. 95:102011. doi: 10.1016/j.learninstruc.2024.102011

[ref45] Tam C. O. (2018). Evaluating students’ performance in responding to art: the development and validation of an art criticism assessment rubric. Int. J. Art Des. Educ. 37, 519–529. doi: 10.1111/jade.12154

[ref46] Venter J. Coetzee S. A. Schmulian A. (2025). Exploring the use of artificial intelligence (AI) in the delivery of effective feedback. Assess. Eval. High. Educ. 50, 516–536. doi: 10.1080/02602938.2024.2415649

[ref47] Xu J. Wang Y. (2024). Do I need feedback or avoid it in L2 writing? Impacts of self-efficacy and shyness on feedback-seeking behavior. Assess. Writ. 59:100805. doi: 10.1016/j.asw.2023.100805

[ref48] Yildiz Durak H. Onan A. (2025). A systematic review of AI-based feedback in educational settings. J. Comput. Soc. Sci. 8:96. doi: 10.1007/s42001-025-00428-1

[ref49] Yuan B. Hu J. (2025). “Generative AI as a tool for enhancing reflective learning in students,” in 2025 IEEE International Conference on Teaching, Assessment, and Learning for Engineering (TALE), (Macao, China: IEEE), 1–6.

[ref50] Zhan Y. Boud D. Dawson P. Yan Z. (2025). Generative artificial intelligence as an enabler of student feedback engagement: a framework. High. Educ. Res. Dev. 44, 1289–1304. doi: 10.1080/07294360.2025.2476513

[ref51] Zhan Y. Yan Z. (2026). Students’ engagement with ChatGPT feedback: implications for student feedback literacy in the context of generative artificial intelligence. Assess. Eval. High. Educ., 51, 821–834. doi: 10.1080/02602938.2025.2471821

