# Supplementary material for: AI feedback-seeking and learning engagement in art education: psychological antecedents and reflective mechanisms
Source: Front Psychol. 2026 Oct 1;17:1948880. doi: 10.3389/fpsyg.2026.1948880 (PMC13668861; doi:10.3389/fpsyg.2026.1948880)
Supplement: Supplementary file 1 [file Supplementary_file_1.DOCX]

Supplementary Material

**Table A.** CMB assessment using the marker-variable technique (Study 1).

| Path | *β*-value | *LLCI* | *ULCI* | *p*-value | Original *R²* | *R²* with marker variable | *\|Δ R²\|* |
| --- | --- | --- | --- | --- | --- | --- | --- |
| MV → AIFS | -0.089 | -0.226 | 0.195 | 0.422 | 0.209 | 0.217 | 0.008 |
| MV → IHA | -0.029 | -0.167 | 0.147 | 0.720 | 0.231 | 0.232 | 0.001 |
| *MV* Marker variable, *AIFS* AI feedback-seeking, *IHA* Interpersonal help-seeking avoidance. | | | | | | | |

**Table B.** The factor loadings, reliability, and convergent validity results of this study (Study 1).

| Construct | Item | Cronbach's α | CR | AVE | Loading |
| --- | --- | --- | --- | --- | --- |
| AI feedback-seeking (AIFS) | AIFS1 | 0.901 | 0.924 | 0.672 | 0.799 |
|  | AIFS2 |  |  |  | 0.815 |
|  | AIFS3 |  |  |  | 0.896 |
|  | AIFS4 |  |  |  | 0.853 |
|  | AIFS5 |  |  |  | 0.836 |
|  | AIFS6 |  |  |  | 0.705 |
| Fear of negative evaluation (FNE) | FNE1 | 0.934 | 0.943 | 0.578 | 0.766 |
|  | FNE2 |  |  |  | 0.788 |
|  | FNE3 |  |  |  | 0.748 |
|  | FNE4 |  |  |  | 0.761 |
|  | FNE5 |  |  |  | 0.742 |
|  | FNE6 |  |  |  | 0.739 |
|  | FNE7 |  |  |  | 0.774 |
|  | FNE8 |  |  |  | 0.764 |
|  | FNE9 |  |  |  | 0.741 |
|  | FNE10 |  |  |  | 0.787 |
|  | FNE11 |  |  |  | 0.807 |
|  | FNE12 |  |  |  | 0.703 |
| Interpersonal help-seeking avoidance (IHA) | IHA1 | 0.944 | 0.953 | 0.691 | 0.859 |
|  | IHA2 |  |  |  | 0.834 |
|  | IHA3 |  |  |  | 0.855 |
|  | IHA4 |  |  |  | 0.803 |
|  | IHA5 |  |  |  | 0.810 |
|  | IHA6 |  |  |  | 0.836 |
|  | IHA7 |  |  |  | 0.843 |
|  | IHA8 |  |  |  | 0.793 |
|  | IHA9 |  |  |  | 0.847 |

**Table C.** Descriptive statistics and discriminant validity results (Study 1).

| Construct | Mean | SD | Skewness | Kurtosis | Fornell-larcker criterion | | |  | HTMT ratio | | |
| --- | --- | --- | --- | --- | --- | --- | --- | --- | --- | --- | --- |
|  |  |  |  |  | 1 | 2 | 3 |  | 1 | 2 | 3 |
| 1.AIFS | 3.774 | 1.347 | 0.101 | -1.151 | **0.820** |  |  |  | / |  |  |
| 2.FNE | 3.952 | 1.271 | -0.041 | -1.188 | 0.256 | **0.760** |  |  | 0.259 |  |  |
| 3.IHA | 3.787 | 1.425 | 0.090 | -1.381 | 0.449 | 0.480 | **0.831** |  | 0.484 | 0.491 | / |
| The bold elements are the square roots of AVE. *AIFS* AI feedback-seeking, *FNE* Fear of negative evaluation, *IHA* Interpersonal help-seeking avoidance. *HTMT* heterotrait–monotrait ratio. | | | | | | | | | | | |

**Table D.** Measurement items of Study 1.

| Construct | Code | Item |
| --- | --- | --- |
| Fear of negative evaluation (FNE) | FNE1 | I worry about what other people think of my artistic performance or work even when I know their opinions make no practical difference. |
|  | FNE2 | I am unconcerned even if I know people are forming an unfavorable impression of my artistic performance or work. |
|  | FNE3 | I am frequently afraid that others will notice shortcomings in my art learning, performance, or work. |
|  | FNE4 | I rarely worry about the impression I make during art learning or presentations of my work. |
|  | FNE5 | I am afraid that teachers, classmates, or others will not approve of my artistic performance or work. |
|  | FNE6 | I am afraid that people will find fault with my art learning or work. |
|  | FNE7 | Other people’s opinions of my artistic performance or work do not bother me. |
|  | FNE8 | When I discuss my art learning or work with teachers or classmates, I worry about what they may think of me. |
|  | FNE9 | I am usually worried about the impression I make during art learning or presentations of my work. |
|  | FNE10 | If I know that teachers or classmates are evaluating me, it has little effect on me. |
|  | FNE11 | Sometimes I think I am too concerned about what others think of my artistic performance or work. |
|  | FNE12 | During art learning or discussions of creative work, I often worry that I will say or do the wrong thing. |
| Interpersonal help-seeking avoidance (IHA) | IHA1 | I do not ask others for help even when an art-learning or creative task is too difficult to solve on my own. |
|  | IHA2 | If I need help to complete an art-learning or creative task, I prefer to skip the difficult part rather than ask others for help. |
|  | IHA3 | I do not ask others for help even when I do not understand the content of an art course. |
|  | IHA4 | If I do not understand something in an art course or creative task, I would rather guess than ask someone for help. |
|  | IHA5 | I would rather perform poorly on an art assignment I cannot finish on my own than ask others for help. |
|  | IHA6 | Even if an art-learning or creative task is too difficult to complete on my own, I would not ask others for help. |
|  | IHA7 | I would rather give any answer or solution than ask others for help. |
|  | IHA8 | I do not ask teachers or classmates questions even when I do not understand an art course or creative task. |
|  | IHA9 | If an art-learning or creative task is too difficult, I would rather not do it than ask others for help. |
| AI feedback-seeking (AIFS) | AIFS1 | When I do not understand the AI’s feedback on my art learning, creative idea, or work, I ask the AI to clarify it. |
|  | AIFS2 | When I do not understand the AI’s feedback on my art learning, creative idea, or work, I ignore it. |
|  | AIFS3 | I ask the AI to tell me what is wrong with my art learning, creative idea, or work. |
|  | AIFS4 | I ask the AI to tell me how I can improve my art learning, creative idea, or work. |
|  | AIFS5 | I ask the AI to point out weaknesses in my art learning, creative idea, or work. |
|  | AIFS6 | I ask the AI to provide specific strategies for improving my art learning, creative idea, or work. |
| Marker variable (MV) | MV1 | Blue is a lovely color. |
|  | MV2 | Blue is a pleasant color. |
|  | MV3 | Blue is a nice color. |
|  | MV4 | I regularly organize my personal belongings.I think blue is a pretty color. |

**Table E.** Inner VIF and *f^2^* values of the model.

| Path | VIF | *f^2^* |
| --- | --- | --- |
| Age → AIFS | 1.013 | 0.001 |
| Art-related major → AIFS | 1.013 | 0.006 |
| FNE → AIFS | 1.306 | 0.003 |
| FNE → IHA | 1.000 | 0.300 |
| Gender → AIFS | 1.029 | 0.000 |
| IHA → AIFS | 1.302 | 0.173 |

**Table F.** CMB assessment using the common method factor (Study 2).

| Construct | Indicator | Substantive factor loading(*R_1_*) | *R_1_^2^* | Method factor loading (*R_2_*) | *R_2_^2^* |
| --- | --- | --- | --- | --- | --- |
| AI feedback-seeking (AIFS) | AIFS1 | 0.834*** | 0.696 | -0.007 | 0.000 |
|  | AIFS2 | 0.800*** | 0.640 | 0.259*** | 0.067 |
|  | AIFS3 | 0.825*** | 0.681 | -0.026 | 0.001 |
|  | AIFS4 | 0.780*** | 0.608 | -0.099 | 0.010 |
|  | AIFS5 | 0.773*** | 0.598 | -0.050 | 0.003 |
|  | AIFS6 | 0.836*** | 0.699 | -0.089 | 0.008 |
| Openness (OPEN) | OPEN1 | 0.924*** | 0.854 | -0.073* | 0.005 |
|  | OPEN2 | 0.876*** | 0.767 | 0.013 | 0.000 |
|  | OPEN3 | 0.914*** | 0.835 | 0.059 | 0.003 |
|  | OPEN4 | 0.885*** | 0.783 | 0.001 | 0.000 |
| Reflective learning (RL) | RL1 | 0.879*** | 0.773 | -0.026 | 0.001 |
|  | RL2 | 0.856*** | 0.733 | -0.091 | 0.008 |
|  | RL3 | 0.885*** | 0.783 | 0.097 | 0.009 |
|  | RL4 | 0.884*** | 0.781 | 0.015 | 0.000 |
| Task behavioral engagement (TBE) | TBE1 | 0.842*** | 0.709 | 0.017 | 0.000 |
|  | TBE2 | 0.817*** | 0.667 | 0.209** | 0.044 |
|  | TBE3 | 0.898*** | 0.806 | -0.069 | 0.005 |
|  | TBE4 | 0.875*** | 0.766 | -0.090 | 0.008 |
|  | TBE5 | 0.720*** | 0.518 | -0.067 | 0.004 |
| Average value | | 0.848 | 0.721 | -0.001 | 0.009 |
| **** p* < 0.001, *** p* < 0.01, ** p* < 0.05. | | | | | |

**Table G.** The factor loadings, reliability, and convergent validity results of this study (Study 2).

| Construct | Item | Cronbach's α | CR | AVE | Loading |
| --- | --- | --- | --- | --- | --- |
| AI feedback-seeking (AIFS) | AIFS1 | 0.894 | 0.918 | 0.652 | 0.834 |
|  | AIFS2 |  |  |  | 0.825 |
|  | AIFS3 |  |  |  | 0.824 |
|  | AIFS4 |  |  |  | 0.766 |
|  | AIFS5 |  |  |  | 0.771 |
|  | AIFS6 |  |  |  | 0.823 |
| Openness (OPEN) | OPEN1 | 0.922 | 0.94 | 0.796 | 0.866 |
|  | OPEN2 |  |  |  | 0.867 |
|  | OPEN3 |  |  |  | 0.948 |
|  | OPEN4 |  |  |  | 0.886 |
| Reflective learning (RL) | RL1 | 0.899 | 0.929 | 0.767 | 0.879 |
|  | RL2 |  |  |  | 0.849 |
|  | RL3 |  |  |  | 0.891 |
|  | RL4 |  |  |  | 0.883 |
| Task behavioral engagement (TBE) | TBE1 | 0.888 | 0.918 | 0.692 | 0.848 |
|  | TBE2 |  |  |  | 0.839 |
|  | TBE3 |  |  |  | 0.888 |
|  | TBE4 |  |  |  | 0.860 |
|  | TBE5 |  |  |  | 0.712 |

**Table H.** Descriptive statistics and discriminant validity results (Study 2).

| Construct | Mean | SD | Skewness | Kurtosis | Fornell-larcker criterion | | | |  | HTMT ratio | | | |
| --- | --- | --- | --- | --- | --- | --- | --- | --- | --- | --- | --- | --- | --- |
|  |  |  |  |  | 1 | 2 | 3 | 4 |  | 1 | 2 | 3 | 4 |
| 1.AIFS | 3.923 | 1.342 | 0.000 | -1.080 | **0.807** |  |  |  |  | / |  |  |  |
| 2.OPEN | 3.660 | 1.643 | -0.003 | -1.559 | -0.086 | **0.892** |  |  |  | 0.118 |  |  |  |
| 3.RL | 3.568 | 1.619 | 0.198 | -1.397 | 0.480 | 0.139 | **0.876** |  |  | 0.522 | 0.122 |  |  |
| 4.TBE | 3.558 | 1.373 | 0.284 | -1.061 | 0.237 | 0.220 | 0.472 | **0.832** |  | 0.244 | 0.221 | 0.520 | / |
| The bold elements are the square roots of AVE. *AIFS* AI feedback-seeking, *OPEN* Openness, *RL* Reflective learning, *TBE* Task behavioral engagement. *HTMT* heterotrait–monotrait ratio. | | | | | | | | | | | | | |

**Table I.** Measurement items of Study 2.

| Construct | Code | Item |
| --- | --- | --- |
| Openness (OPEN) | OPEN1 | I have a vivid imagination. |
|  | OPEN2 | I am not interested in abstract ideas. |
|  | OPEN3 | I have difficulty understanding abstract ideas. |
|  | OPEN4 | I do not have a good imagination. |
| AI feedback-seeking (AIFS) | AIFS1 | When I did not understand the AI’s feedback on my creative idea or proposal, I asked the AI to clarify it. |
|  | AIFS2 | When I did not understand the AI’s feedback on my creative idea or proposal, I ignored it. |
|  | AIFS3 | I asked the AI to tell me what was problematic in my creative idea or proposal. |
|  | AIFS4 | I asked the AI to tell me how I could improve my creative idea or proposal. |
|  | AIFS5 | I asked the AI to point out weaknesses in my creative idea or proposal. |
|  | AIFS6 | I asked the AI to provide specific strategies for improving my creative idea or proposal. |
| Reflective learning (RL) | RL1 | While completing the task, I sometimes questioned existing ways of doing things and tried to think of a better way. |
|  | RL2 | I thought over what I had been doing and considered alternative ways of doing it. |
|  | RL3 | I reflected on my actions during the task to see whether I could have improved what I did. |
|  | RL4 | I re-appraised my task experience so that I could learn from it and improve my next performance. |
| Task behavioral engagemen (TBE) | TBE1 | I tried hard to do well on this art-creation task. |
|  | TBE2 | While using AI to refine the proposal, I worked as hard as I could. |
|  | TBE3 | While using AI to refine the proposal, I actively participated in the task activities. |
|  | TBE4 | While using AI to refine the proposal, I stayed focused on the task. |
|  | TBE5 | While using AI to refine the proposal, I carefully considered the task requirements and possible improvements. |

**Table J.** Inner VIF and *f^2^* values of the model.

| Path | VIF | *f^2^* |
| --- | --- | --- |
| Age → TBE | 1.034 | 0.017 |
| AI Familiarity →TBE | 1.029 | 0.005 |
| AIFS → RL | 1.007 | 0.391 |
| AIFS →TBE | 1.304 | 0.000 |
| Gender → TBE | 1.029 | 0.011 |
| OPEN → RL | 1.016 | 0.036 |
| OPEN × AIFS → RL | 1.009 | 0.161 |
| RL → TBE | 1.311 | 0.220 |
